# Full-Length Transcriptome Sequencing and Comparative Transcriptomics Reveal the Molecular Mechanisms Underlying Gonadal Development in Sleepy Cod (*Oxyeleotris lineolata*)

**DOI:** 10.3390/biology14030232

**Published:** 2025-02-25

**Authors:** Jiajia Fan, Dongmei Ma, Huaping Zhu, Minghui Lin, Zaixuan Zhong, Yuanyuan Tian

**Affiliations:** 1 Key Laboratory of Tropical & Subtropical Fishery Resource Application & Cultivation, Ministry of Agriculture and Rural Affairs, Pearl River Fisheries Research Institute, Chinese Academy of Fishery Sciences, Guangzhou 510380, China; fjj@prfri.ac.cn (J.F.); zhhping2000@163.com (H.Z.); linmmh81@163.com (M.L.); zhongzaixuan0429@126.com (Z.Z.); tianyuan320@163.com (Y.T.); 2 Key Laboratory of Aquatic Animal Immunology and Sustainable Aquaculture, Pearl River Fisheries Research Institute, Chinese Academy of Fishery Sciences, Guangzhou 510380, China

**Keywords:** sleepy cod Oxyeleotris lineolata, sexual dimorphism, transcriptome, sex determination, gonadal differentiation

## Abstract

Sleepy cod (*Oxyeleotris lineolata*) is native to Australia and is now an economically valuable fish cultured in China and Southern Asian countries. Its growth rate exhibits as sexually dimorphic, with males generally growing more rapidly and attaining a larger body size compared to females. Thus, the effective development of sex control breeding can significantly contribute to increased yields and output value. Nevertheless, due to the lack of genomic and transcriptomic data, the molecular mechanisms underlying sex determination and gonadal differentiation in sleepy cod remain poorly understood. In this study, long-read PacBio isoform sequencing (Iso-Seq) was performed to obtain a full-length transcriptome from a pooled sample of eight tissues (kidney, brain, liver, muscle, heart, spleen, ovary and testis). A total of 30.41 G subread bases were generated and 49,113 non-redundant full-length transcripts with an average length of 2948 bp were produced. Using the full-length transcriptome as a reference, short-read Illumina sequencing was performed to investigate the differences in gene expression at the transcriptome level between ovaries and testes from 12-month-old individuals. A total of 19,102 differentially expressed transcripts (DETs) were identified, of which 8510 (44.55%) were up-regulated in the ovary and 10,592 (55.45%) were up-regulated in the testis. The DETs were mainly clustered into 241 KEGG pathways, in which oocyte meiosis and arachidonic acid metabolism were the most relevant pathways involved in gonadal differentiation. To verify the validity of the transcriptomic data, 20 DETs were selected to investigate the gonad expression profiles based on qPCR. The expression levels of all 20 screened genes were consistent with the transcriptome sequencing results. The present study provides new genetic resources—including full-length transcriptome sequences and annotation information—as a coding genomic-level reference for sleepy cod—yielding valuable insights into the genetic mechanisms of sex determination and gonadal differentiation in this economically important species.

## Simple Summary

The sleepy cod *Oxyeleotris lineolata* is a species of freshwater goby, which is natively distributed throughout northern Australia. Due to its tender meat and delicious taste, it has become a freshwater fish of economic importance, with a relatively high price in Asian markets. We observed that sleepy cod presents as sexually dimorphic in its growth rate and body size, with the average body weight of mature males being significantly higher than that of mature females (*p* < 0.05). Therefore, the efficient development of all-male breeding will be helpful in increasing the yield and output value of this species. In this context, it is important to obtain more genetic information to understand the sex determination and gonadal differentiation mechanism of sleepy cod. The present study provides new genetic resources—including full-length transcriptome sequences and annotation information—as a coding genomic-level reference for sleepy cod—yielding valuable insights into the genetic mechanisms of sex determination and gonadal differentiation in this economically important species.

## 1. Introduction

### 1.1. Sexual Dimorphism in Fish

Teleosts are the largest and most diverse group of vertebrates, with about 27,000 species [1], accounting for more than 50% of all known vertebrate species. In contrast to mammals and birds, the sex determination and differentiation mechanisms of teleosts are primitive, diverse and changeable, and almost all known sex determination types of vertebrates have been found in fish [2,3,4]. Most teleosts present as sexually dimorphic in multiple traits (e.g., morphology, growth rate and physiology) and, so, sex control breeding has become a hotspot of genetic and breeding research in fish. Exploring the mechanisms of sex determination and gonad differentiation in fish can help in sex control, allowing for all-female or all-male breeding [5]. Obviously, monosex fish breeding has potential advantages, such as achieving a higher average growth rate, elimination reproduction, reductions in territorial behaviors, minimizing size variations at harvest and lowering the environmental risks posed by exotic species escapes [6]. In recent years, sex control breeding techniques have been applied in many economic fish species, including *Oreochromis niloticus* [7], *Pelteobagrus fulvidraco* [8] and *Cyprinus carpio* [9], among others.

### 1.2. Introduction to the Sleepy Cod

The sleepy cod *Oxyeleotris lineolata* is a species of freshwater goby, which is natively distributed throughout northern Australia [10]. At present, it is widely cultured in reservoirs, lakes and rivers as well as ponds in Australia and Southeast Asia [11]. Due to its tender meat and delicious taste, it has become a freshwater fish of economic importance, with a relatively high price in Asian markets [12]. While there have been a few related studies on sleepy cod, they have mainly focused on its reproductive physiology [10,11], mitochondrial sequence [13] and SNP (single nucleotide polymorphism) screening [14]. Sleepy cod reach sexual maturity in two years. In the breeding season, females have a broad, flattened genital papilla, different from the triangular one of males and juveniles. Females can spawn up to 10 times per breeding season. Eggs are usually laid under surfaces. Most spawning happens between 05:00 and 10:00 h. The average number of eggs per spawn is 43,130 [10,11]. We observed that sleepy cod presents as sexually dimorphic in its growth rate and body size, with the average body weight of mature males being significantly higher than that of mature females (*p* < 0.05). Therefore, the efficient development of all-male breeding will be helpful in increasing the yield and output value of this species. In this context, it is important to obtain more genetic information to understand the sex determination and gonadal differentiation mechanisms of sleepy cod.

### 1.3. Introduction to the RNA Sequencing

RNA sequencing (RNA-seq) is an extremely powerful tool for revealing genotype–phenotype connections. It also allows for a deeper comprehension of the underlying pathways and molecular mechanisms that regulate development, growth, immune regulation and so on. However, second-generation sequencing technology has its drawbacks, such as generating only short reads and having an amplification bias, which restricts the ability to obtain full-length transcripts and high-quality reference sequences [15]. Thus, the Pacific Biosciences (PacBio) RNA sequencing solution—called the long-read isoform sequencing (Iso-seq) method—makes use of single-molecule real-time (SMRT) sequencing technology. This technology produces full-length and highly accurate long reads. Nevertheless, the expense associated with Iso-seq is significantly greater than that of second-generation sequencing. As a result, these long reads can be utilized in conjunction with short-read RNA-seq techniques to overcome the limitations of second-generation sequencing [16,17]. Full-length transcriptomes with complete coding sequences allowing for the characterization of gene families and have been obtained for many teleost species, such as *Acipenser schrenckii* [18], *Salvelinus malma* [19], *Misgurnus anguillicaudatus* [20] and *Danio rerio* [21]. The full-length transcriptome can serve as a reliable reference for gene expression analysis of RNA-seq data [22]. Therefore, in the present study, a high-quality reference transcriptome of sleepy cod was constructed via SMRT sequencing. Then, RNA-seq was performed on sleepy cod testes and ovaries to characterize key pathways and genes that are specifically active in the gonads.

## 2. Materials and Methods

### 2.1. Ethics Statement

In this study, all experiments were conducted in accordance with the recommendations in the Guide for the Care and Use of Laboratory animals of Pearl River Fishery Research Institute (Guangzhou, China). The protocols for fish handling and sampling were approved by the Committee on the Ethics of Animal Experiments of Pearl River Fishery Research Institute (LAEC-PRFRI-2023-03-31).

### 2.2. Experimental Samples

Sleepy cods were obtained from the Guangzhou Ruifeng Fishery Development Limited Company, Guangzhou, China. A total of 20 adults (12 females and 8 males) were selected as parents for propagation and breeding. Offspring were cultured for 12 months in a pond at a density of 1000 fry per 667 m^2^. Among them, a total of 92 fish were randomly selected. The fish were anesthetized in water at 28 °C with Tricaine (MS-222) at a concentration of 100 mg/L, and then their growth traits (body weight, body length, body depth and body width) were measured and their genders were identified. Body weight was measured using an electronic balance (precision of 0.01 g), and the body length, body depth and body width were measured using a Vernier caliper (precision of 0.01 cm). All data are presented as mean ± standard deviation (SD). All of the 92 fish were dissected for determination of gender. Statistical analysis was performed via one-way ANOVA with the SAS software (version 9.4) (SAS Institute Inc., Cary, NC, USA).

Next, 8 tissues (gonad, brain, kidney, liver, muscle, heart, intestine and spleen) were sampled separately from male (*n* = 3) and female (*n* = 3) sleepy cod. Total RNA extracted from these samples was used to construct PacBio libraries and Illumina libraries and to perform quantitative real-time PCR (qPCR). The ovaries (*n* = 3) and testes (*n* = 3) were fixed in Bouin’s solution for histological observation.

### 2.3. Histological Analysis of the Gonads

After dehydration and paraffin embedding, the fixed ovaries and testes were sectioned at 6 μm using a microtome (Leica RM2235, Wetzlar, Germany). Then, the sections were stained with hematoxylin and eosin (HE) and observed under a microscope (Leica DM750, Germany).

### 2.4. RNA Extraction and Quality Evaluation

For each sample, total RNA was extracted using Trizol Reagent (Invitrogen, Waltham, MA, USA). Subsequently, genomic DNA was eliminated with the help of gDNA eraser (TaKaRa, Dalian, China). The purity and concentration of RNA samples were determined through a combination of a Nanodrop 2100 spectrophotometer (Thermo Scientific, Waltham, MA, USA) and 0.8% agarose gel electrophoresis. RNA samples with an OD_260/280_ ratio within the range of 1.8 to 2.2 were selected. RNA integrity was assessed using an Agilent 2100 Bioanalyzer System (Agilent Technologies, Santa Clara, CA, USA), and only those with an RNA Integrity Number (RIN) score of 8.0 or higher met the criteria.

### 2.5. Library Construction for PacBio and Illumina Sequencing

In order to obtain the full-length transcriptome of sleepy cod, 8 tissue types, namely the ovary, testis, brain, kidney, liver, muscle, heart, intestine and spleen, were chosen. A precise quantity of 1.0 µg of total RNA was isolated from each individual tissue and then mixed. Total RNA (8.0 µg) was reversely transcribed into cDNA using a SMARTer^TM^ PCR cDNA Synthesis Kit (Clontech, Mountain View, CA, USA), in order to construct one single-molecule real-time (SMRT) library. The SMRT library was sequenced using the PacBio Iso-seq system by Biomarker Technologies Co., Ltd. (Beijing, China). Short-read RNA-seq, which is known for its advantages such as high throughput, high accuracy, and cost-effectiveness, was employed in this study. RNA samples were separately collected from 3 ovaries and 3 testes for short-read RNA-seq analysis. The RNA-seq transcriptome libraries were prepared using a TruSeq^TM^ RNA sample preparation kit from Illumina (San Diego, CA, USA). Then, these libraries were sequenced on the Illumina HiSeq 2500 platform and 150 bp paired-end reads were generated.

### 2.6. Processing of Sequencing Data

The raw PacBio Iso-seq data were analyzed using the SMRTlink v7.0 software (https://www.pacb.com/products-and-services/analytical-software/smrt-analysis/, accessed on 25 March 2020). The circular consensus sequence (CCS) was generated from the subread BAM files, also known as the reads of insert (ROI). Sequencing reads having full passes of 2 or more were chosen and utilized to extract ROIs. These ROIs were then categorized into two types: full-length non-chimeric (FLNC) transcripts and non-full length (nFL) transcripts. Similar FLNC transcripts were clustered hierarchically using Minimap2 [23], following which the consensus transcripts were polished and filtered using the Quiver software with a criteria of post-correction accuracy above 99% and the CD-HIT program (version 4.8.1) with a threshold of 0.99 identity [24]. High-accuracy FLNC transcripts were obtained as a high-quality reference transcriptome database.

We used FastQC (http://www.bioinformatics.babraham.ac.uk/projects/fastqc/, accessed on 25 March 2020) and TrimGalore (https://github.com/FelixKrueger/TrimGalore, accessed on 25 March 2020) for quality control of RNA—seq short reads, filtering out low-quality data (adapter—containing, <50 bp, Q < 20). The filtered reads were then mapped to high-accuracy FLNC transcripts in the full-length transcriptome of sleepy cod using the Tophat2 tool.

### 2.7. Functional Annotation of Transcripts

To annotate all transcripts, we used the BLAST software (version 2.2.26) (https://blast.ncbi.nlm.nih.gov/Blast.cgi, accessed on 25 March 2020) [25] to compare them with seven public databases, analyzing with BLAST at an E-value < 10^−10^. These databases included NCBI Non-redundant Protein Sequences (Nr; https://www.ncbi.nlm.nih.gov/protein/, accessed on 25 March 2020) [26], Cluster of Orthologous Groups of proteins (COG; http://www.ncbi.nlm.nih.gov/COG, accessed on 25 March 2020) [27], Protein Family (Pfam; http://www.ncbi.nlm.nih.gov/COG) [28], Swiss Protein Data Bank (Swiss-Prot; https://www.uniprot.org/help/uniprotkb_swissprot, accessed on 25 March 2020) [29], Kyoto Encyclopedia of Genes and Genomes (KEGG; http://www.genome.ad.jp/kegg/) [30], Gene Ontology (GO; http://www.geneontology.org, accessed on 25 March 2020) [31] and Evolutionary Genealogy of Genes: Non-supervised Orthologous Groups (eggNOG; http://eggnog.embl.de, accessed on 25 March 2020) [32].

### 2.8. Differential Expression Analysis of Ovary and Testis

For further analysis, the expression levels of genes were compared between the ovaries and testes, and the high-quality short-reads generated through RNA-seq were mapped back to the reference sequences derived from PacBio Iso-seq. The RSEM software (version 1.3.3) (http://deweylab.biostat.wisc.edu/rsem, accessed on 25 March 2020) [33] was employed to obtain and normalize both the counts of the mapped reads and the Fragments Per Kilobase of transcript per Million mapped reads (FPKM). Before performing the differential gene expression analysis, the read counts were refined using the edgeR package. This refinement was achieved through applying a single scaling normalization factor for each of the sequenced libraries. Differentially expressed transcripts (DETs) were analyzed between ovaries and testes using the DESeq2 software (version 1.26.0) (https://bioconductor.org/packages/release/bioc/html/DESeq2.html, accessed on 25 March 2020) [34]. The false discovery rate (FDR) was calculated using the posterior probability of differential expression (PPDE). FDR < 0.01 and fold change ≥ 2 were set as the threshold for DETs.

### 2.9. Functional Enrichment Analysis of DETs

The GO enrichment analysis of the DETs was performed using the GOseq R packages. These packages are based on Wallenius non-central hyper-geometric distribution [35] and are capable of correcting for gene length bias in DETs. GO terms with a corrected *p*-value less than 0.05 were regarded as being significantly enriched in the DETs. Furthermore, a KEGG pathway analysis was carried out to test the statistical enrichment of differentially expressed transcripts using the KOBAS software (version 2.0) [36].

### 2.10. Protein–Protein Interaction (PPI) Network Construction

A PPI network was constructed using STRING v12.0 (https://string-db.org/, accessed on 25 March 2020) with default parameters, using the *Danio rerio* database. The PPI networks were visualized using the Cytoscape software (version 3.4.0).

### 2.11. Quantitative Real-Time PCR (qPCR)

To further validate the confidence of the RNA-seq data, 20 DETs were selected and detected by qPCR. Specific primers were designed based on the reference sequences within the full-length transcriptome of sleepy cod (Table 1). The *β-actin* gene was selected as the internal reference. Total RNA was extracted from ovaries (*n* = 3) and testes (*n* = 3). Then, a PrimeScript™ RT reagent Kit with gDNA Eraser (Takara, Beijing, China) was employed to synthesize the first-strand cDNA.

Subsequently, qPCR assays were conducted using the SYBR Premix Ex TaqII (ABI, Foster, CA, USA) on a 7500 Real-Time PCR System (ABI, USA). The reaction volume was set at 20 μL, which consisted of 10 μL of 2 × Master Mix, 0.4 μL of both the forward and reverse primers (10 μmol/L) and 1 μL of the synthesized cDNA, and the rest was made up with ddH_2_O. The reaction conditions were precisely defined as follows: an initial pre-denaturation step at 95 °C for 2 min, followed by 40 cycles. Each cycle included a denaturation phase at 95 °C for 5 s, an annealing step at 60 °C for 31 s and an extension phase at 72 °C for 30 s. After the 40 cycles, a final extension at 72 °C for 10 min was carried out, following which a melting curve analysis was performed with conditions of 95 °C for 15 s, 60 °C for 30 s and 95 °C for 15 s. Finally, the relative expression fold changes of 20 genes in female and male tissues were analyzed by applying the 2^−ΔΔCt^ method [37].

## 3. Results

### 3.1. Differences in Growth Traits Between Female and Male of Sleepy Cod

A total of 92 sleepy cod individuals (131.30–199.98 g) were dissected for examination of gender. The sex ratio was close to 1:1 (48 females and 44 males). The body weight of females was 144.85 ± 33.10 g, while that of males was 185.80 ± 57.29 g, with the mean of males being 1.28 times higher than that of the females. The body length of females was 18.76 ± 1.72 cm and that of males was 20.93 ± 1.96 cm, with the mean of males being 1.16 times larger than that of the females. Furthermore, the mean body depth and body width of the males were larger than those of the females. Overall, there were significant differences in body weight, body length, body depth and body width between the females and males (*p* < 0.01), according to the one-way analysis of variance (ANOVA; see Table 2). Prior to conducting the ANOVA, the Shapiro–Wilk test was employed to check the normality of the data, and the T-test within the ANOVA was used to assess the significance of the differences.

### 3.2. Histological Characteristics of the Sleepy Cod Gonads

**Figure 1:**
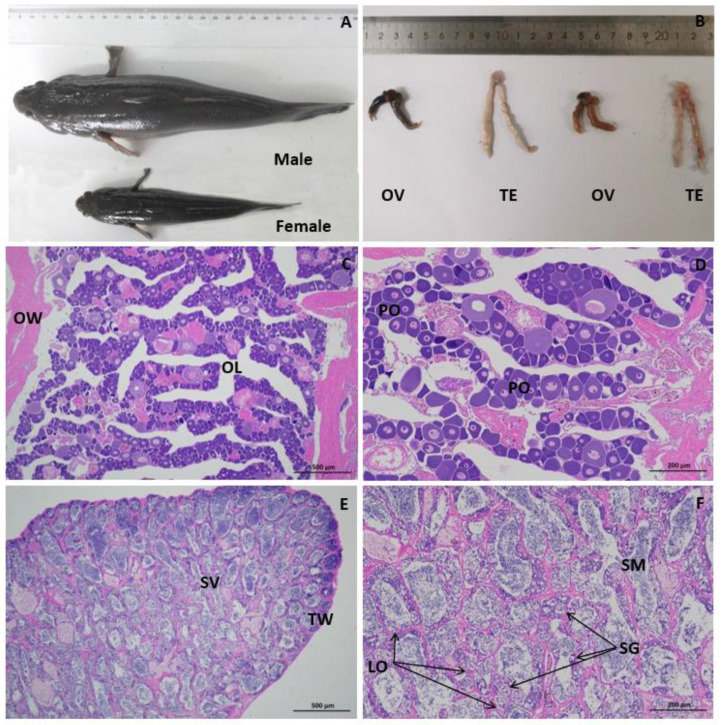
Morphology and histological characteristics of sleepy cod gonads: (**A**) The morphology of 12-month-old male and female individuals (The interval between adjacent data points is 1 cm); (**B**) The morphology of the ovaries (OV) and testes (TE); (**C**) Histological characteristics of ovary (400×); (**D**) Histological characteristics of ovary (1000×); (**E**) Histological characteristics of testes (400×); (**F**) Histological characteristics of testes (1000×); OW, ovary wall; PO, primary oocyte period; OL, ovarian lumen; TW, testis wall; LO, lobule; SV, seminal vesicle; SG, spermatogonia; SM, spermatid. The gonadal tissues were stained with hematoxylin and eosin (HE).

A pair of gonads were observed between the kidney and the digestive tract in the abdominal cavity of every individual (Figure 1). The ovary and testis can be distinguished phenotypically by mere observation. The ovary is gray or flesh-colored, and the egg granules can be clearly observed. Meanwhile, the testis is milky white-colored and flat. Histological analysis showed that most ovaries and testes of the 12-month-old sleepy cod were at stage III of maturity. In the ovaries of these individuals, a significant number of primary oocytes were distinctly observable. Along the nuclear periphery, numerous small nucleoli were present; however, the development of these primary oocytes exhibited asynchrony. The testes consisted of seminiferous lobules. The sex cells consisted of spermatogonia and spermatids (Figure 1).

### 3.3. The Full-Length Transcriptome of Sleepy Cod

PacBio Iso-seq technology was employed to generate the full-length transcriptome of sleepy cod. The pooled RNA used for this purpose was collected from eight different tissues, including ovary, testis, brain, kidney, liver, muscle, heart, intestine and spleen. A total of 30.41 G subread bases were generated with 390,583 CCS numbers. Following the standard Iso-Seq classification and clustering protocol, all the ROIs underwent further categorization. Eventually, 318,398 FLNC reads and 72,185 nFL reads were acquired, with an average length of 2948 bp. Then, 84,085 consensus sequences with average length of 3053 bp were produced using the SMRTlink v5.0 software. Finally, a total of 49,113 non-redundant transcripts were obtained via de-redundancy analysis using the CD-HIT software.

### 3.4. Functional Gene Annotation for Full-Length Transcripts of Sleepy Cod

In order to achieve a thorough functional annotation of the full-length transcriptome of sleepy cod, all 49,113 non-redundant transcripts were annotated using eight distinct databases, including NR, eggNOG, GO, KEGG, Pfam, SwissProt, KOG and COG. A total of 92.65% of the transcripts (45,505 of 49,113) were successfully annotated in at least one database (Table 3). It is highly likely that the remaining 3608 unannotated transcripts could potentially represent novel species-specific genes exclusive to the sleepy cod. This speculation suggests that these transcripts might play crucial roles in the unique biological characteristics and functions of the sleepy cod, which could offer new insights into the evolution, development and physiological processes of the species.

**Figure 2:**
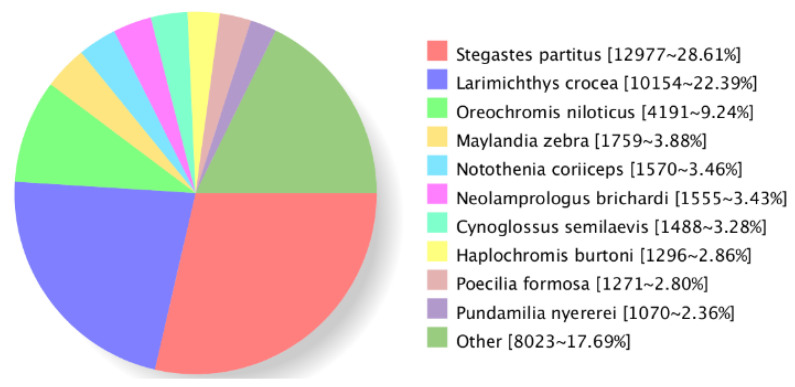
Homologous species distribution of sleepy cod full-length transcripts annotated in the NR database.

**Figure 3:**
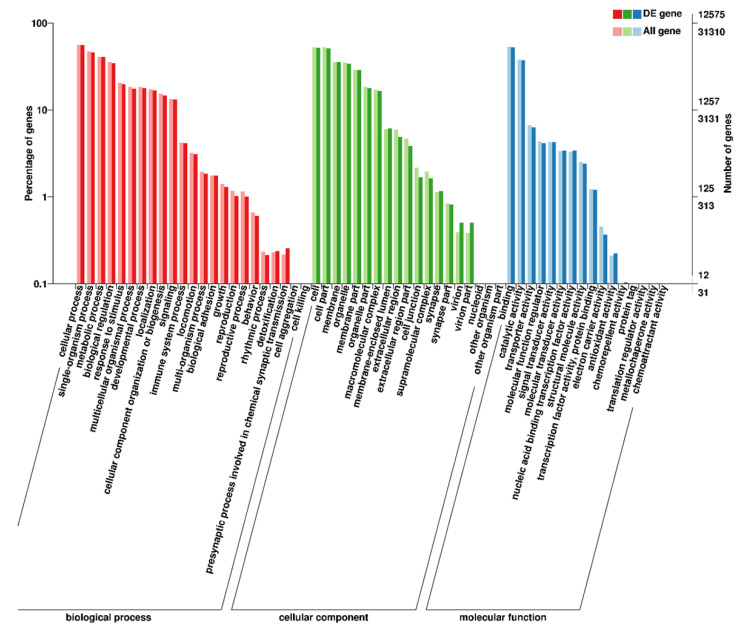
GO term annotation classification statistics for sleepy cod transcripts. The *x*-axis represents GO categories, the *y*-axis (**left**) represents the percentage of transcripts and the *y*-axis (**right**) represents the number of all transcripts (All gene) or DETs (DE genes).

There were 45,316 (92.36%) transcripts matched in the NR database, and the matches showed that 28.61% of transcripts had significant similarity to *Stegastes partitus*, followed by *Larimichthys crocea* (22.39%) and *Oreochromis niloticus* (9.24%); see Figure 2. At the same time, 31,310 (63.75%) transcripts were successfully annotated to the GO database and were classified into three major categories: Biological process (23 terms), cellular component (19 terms) and molecular function (16 terms); see Figure 3. The most-enriched GO terms were “cellular process” in the biological process category, “cell” in the cellular components category and “binding” in the molecular function category. Moreover, two sex-related terms—reproduction (363 transcripts) and reproductive process (362 transcripts)—were also enriched in the biological process category.

Then, the transcripts were aligned to the KEGG database to further classify the biological pathways. There were 30,523 (62.15%) transcripts assigned to 284 pathways (Table S1). Endocytosis (1056 transcripts), herpes simplex infection (805 transcripts) and phagosome (723 transcripts) were the top three pathways with the most abundant transcripts.

### 3.5. Illumina Sequencing and Analysis of Ovary and Testis

The cDNA libraries constructed from three testes and three ovaries of sleepy cod were sequenced using the Illumina platform. Through this sequencing process, a total of 55.23 G raw reads were successfully acquired. The quality evaluation indices of the high-quality short reads are presented in Table 4. In summary, each sample yielded at least 2.62 × 10^7^ high-quality clean short reads, and the Q30 percentage of these reads exceeded 93.14% (Table 4).

### 3.6. DETs in Testes and Ovaries

**Figure 4:**
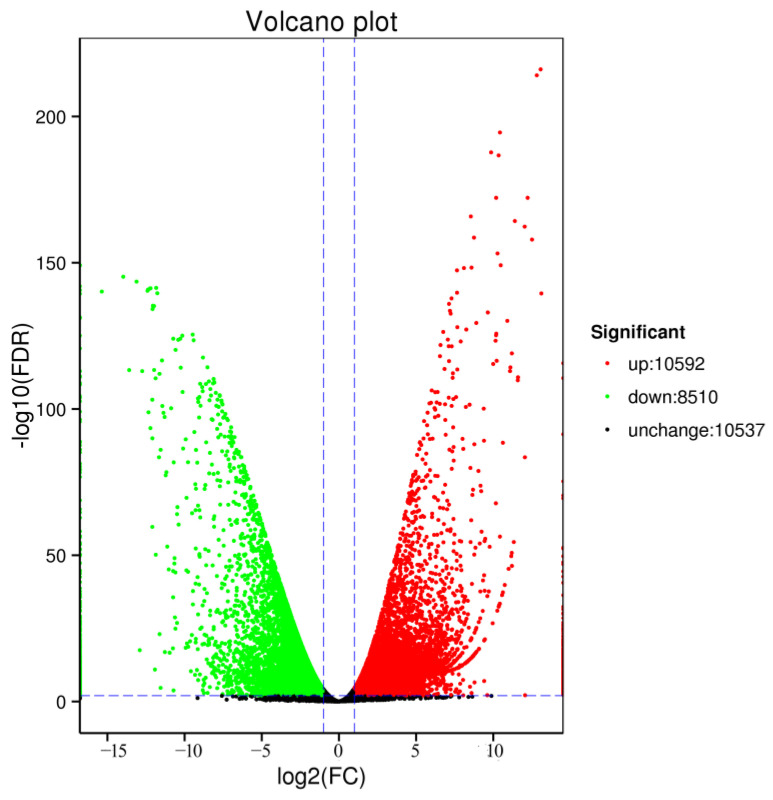
Volcano plot of the DET distribution in the testes and ovaries of sleepy cod. The horizontal axis is log2 Fold change, the vertical axis is log10 *p*-value. Each dot represents one gene. The DETs are marked in red and green.

Using the non-redundant full-length transcripts as references and merging the clean short-read data derived from the Illumina sequencing platform, 29,639 transcripts in testes and ovaries of were obtained. Among them, 18,445 ovary-biased and testis-biased transcripts were assigned using eight databases, including NR, COG, KOG, Pfam, SwissProt, KEGG, GO, and eggNOG (Table 5). On the other hand, the distribution of testis- and ovary-related transcripts is shown as a Volcano plot in Figure 4. A total of 10,592 transcripts (35.74%) showed ovary-biased expression patterns, 8510 transcripts (28.71%) were testis-biased and 10,537 transcripts (35.6%) were shared expression patterns.

A total of 12,575 DETs were annotated in the GO database. These DETs were then classified into three main categories: biological process, which consisted of 23 GO terms; molecular function, with 16 GO terms; cellular component, containing 19 GO terms (Figure 3). We performed molecular function category GO term enrichment analyses for ovary- and testis-biased DETs separately. As shown in Figure S1, we discovered that there were significant differences in the enriched GO terms between the ovary- and testis-biased DETs. For the ovary-biased DETs, the majority of the transcripts were associated with the top three GO terms of DNA helicase activity, ATP binding and 1-SMAD binding. However, most of the testis-biased DETs were related to serine-type endopeptidase activity, calcium ion binding and fructose-bisphosphate aldolase activity.

**Figure 5:**
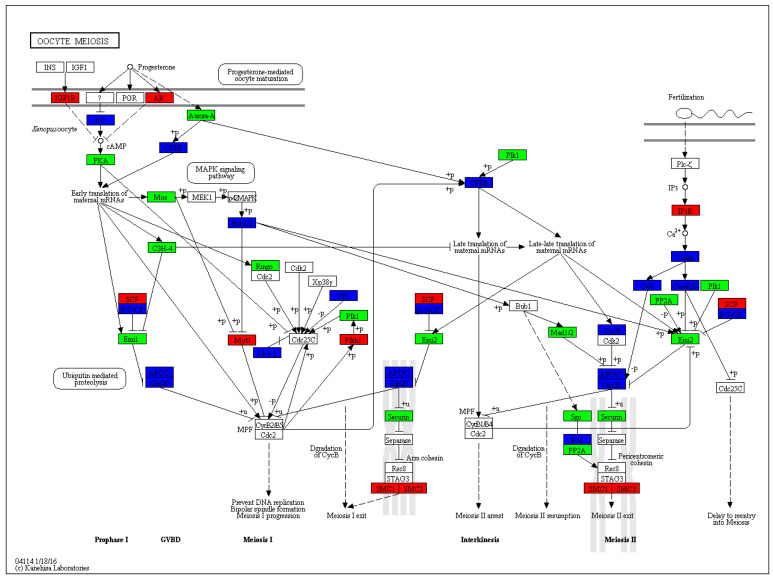
The oocyte meiosis signaling pathway, with genes detected in ovary and testis transcriptomes. Rectangular nodes correspond to genes or gene families and their color indicates mRNA expression (red means up-regulated, green means down-regulated, blue means contains both up-regulated and down-regulated, white means not differentially expressed).

**Figure 6:**
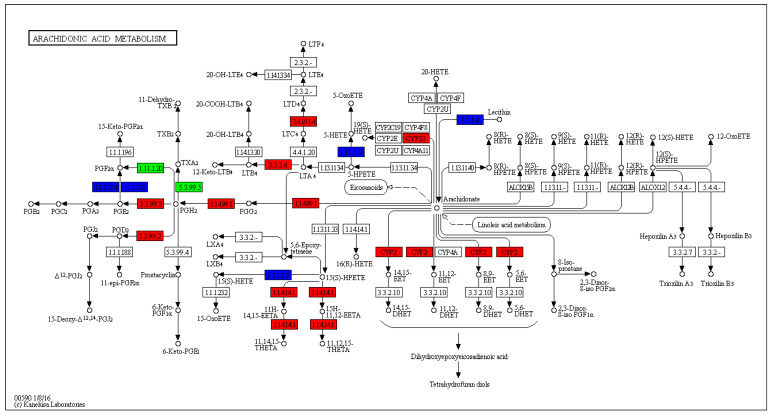
The arachidonic acid metabolism pathway, with genes detected in ovary and testis transcriptomes. Rectangular nodes correspond to genes or gene families and their color indicates mRNA expression (red means up-regulated, green means down-regulated, blue means contains both up-regulated and down-regulated, white means not differentially expressed).

**Figure 7:**
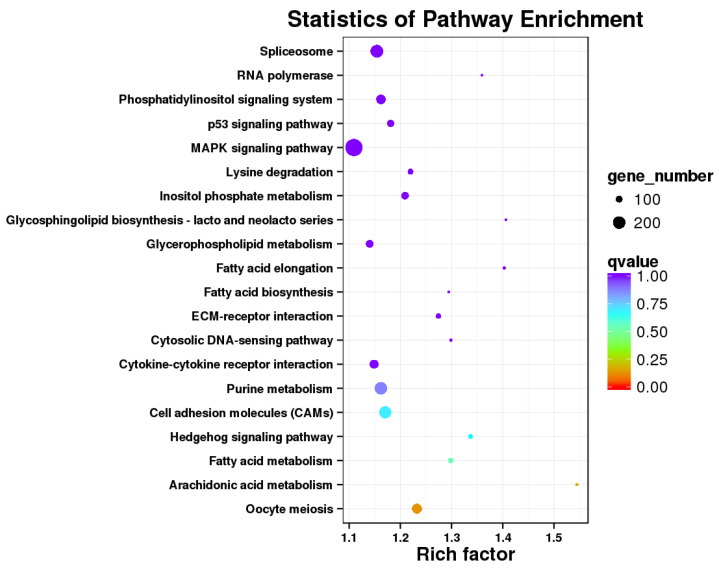
The top 20 KEGG pathways of sex-related DETs.

The 12,099 DETs were annotated in 241 KEGG pathways, but only 23 pathways were significantly different (q-value < 0.05), of which oocyte meiosis (Figure 5) and arachidonic acid metabolism (Figure 6) were the most relevant pathways involved in gonadal differentiation (Figure 7, Table 6, Table S1). We independently carried out KEGG pathway enrichment analysis for the ovary- and testis-biased DETs. As presented in Figure S2, there were notable disparities in the enriched KEGG pathways between the ovary- and testis-biased DETs. For the ovary-biased DETs, the majority of the transcripts were associated with the top three KEGG pathways of DNA replication, cell cycle and RNA degradation. In contrast, the majority of transcripts among the testis-biased DETs were implicated in neuroactive ligand–receptor interaction, cardiac muscle contraction and phagosome.

### 3.7. Validation of Transcriptome Sequencing Results via qPCR

**Figure 8:**
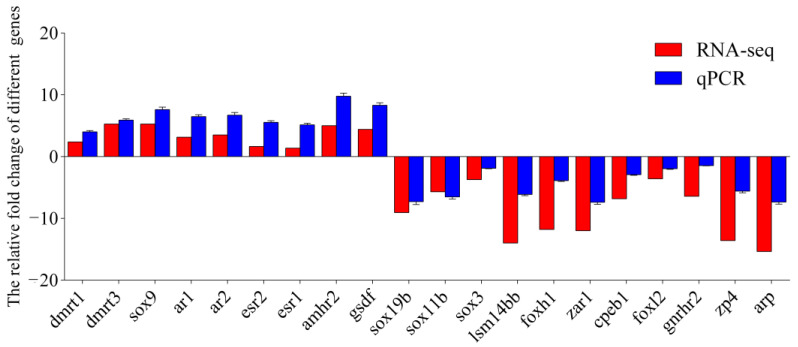
Validation of the expression levels of 9 up-regulated and 11 down-regulated DETs via qPCR. RNA-Seq and qPCR data are indicated in red and blue, respectively. The fold changes of 20 genes in male versus female tissues obtained via RNA-seq were calculated via FPKM. The log2 fold change values of qPCR and RNA-seq for these genes are used for graphical presentation.

To validate the reliability and validity of the transcriptome sequencing (RNA-seq), 20 differentially expressed genes were selected from the DETs for qPCR validation (Figure 8). A total of 11 of these genes were highly expressed in the ovary, including SRY-box transcription factor 19b (*sox19b*), SRY-box transcription factor 11b (*sox11b*), SRY-box transcription factor 3 (*sox3*), LSM family member 14B (*lsm14bb*), forkhead box H1 (*foxh1*), zygote arrest 1 (*zar1*), cytoplasmic polyadenylation element binding protein 1 (*cpeb1*), forkhead box L2 (*foxl2*), Gonadotropin releasing hormone receptor 2 (*gnrhr2*), zona pellucida glycoprotein 4 (*zp4*) and atrophin-1 (*arp*). Additionally, nine genes were highly expressed in the testis, including doublesex- and mab-3-related transcription factor 1 (dmrt1), doublesex- and mab-3-related transcription factor 3 (*dmrt3*), SRY-box transcription factor 9 (*sox9*), androgen receptor-1 (*ar1*), androgen receptor-2 (*ar2*), estrogen receptor-1 (*esr1*), estrogen receptor-2 (*esr2*), anti-Mullerian hormone receptor type-2 (*amhr2*) and gonadal soma derived factor (*gsdf*). Melting curve analysis revealed that primers of all tested genes were specific. The qPCR results were significantly correlated with the RNA-seq results, with a correlation coefficient of 0.943 (*p* < 0.01), indicating the credibility of the RNA-seq data.

**Figure 9:**
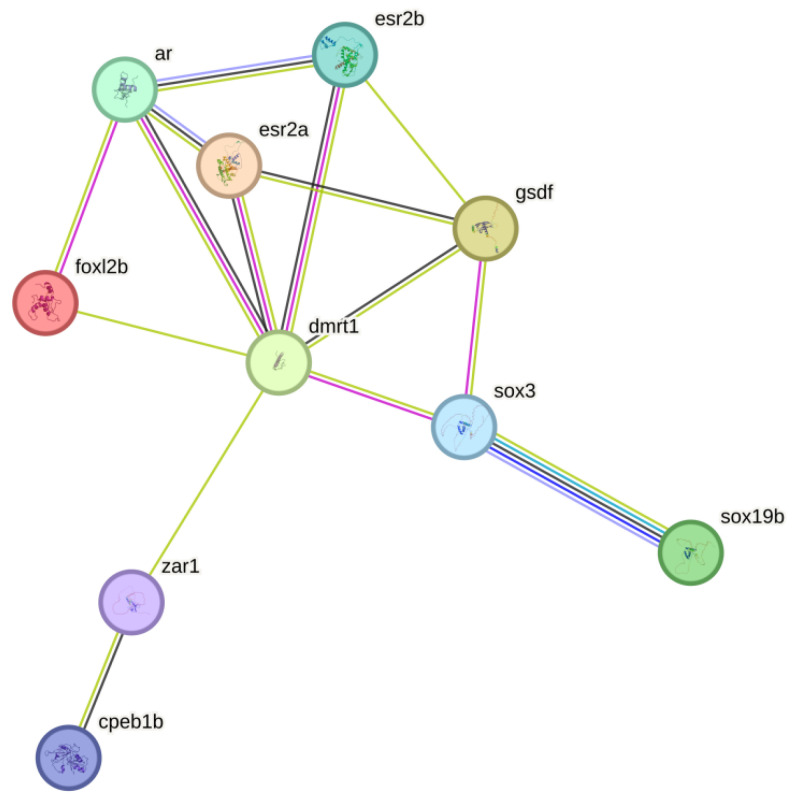
The protein–protein interaction network of 10 DETs from sleepy cod.

In order to gain deeper insight into the molecular mechanisms through which these 20 sex-related genes influence gonadal development, we constructed PPI networks. A total of 10 proteins were involved in the interaction network, and there were 30 interactions; see Figure 9. Notably, the DMRT1 protein has 7 interaction networks, while ar and gsdf each have 4 interaction networks. Furthermore, we employed qPCR to examine the expression patterns of these 20 genes across 8 different tissues (gonad, brain, kidney, liver, muscle, heart, intestine and spleen). Our findings indicated that, aside from their differential expression in the testes and ovaries, *sox3*, *cpeb1*, *foxl2*, *sox9* and *dmrt3* exhibited relatively elevated expression levels within the brain tissue (Figure S3).

## 4. Discussion

Growth performance is an important index for inferring potential success in aquaculture. Being sexually dimorphic in size is both universal and diverse among fish species. In females, larger body sizes are generally advantageous as they are often associated with increased fecundity. Larger females can produce more eggs, thereby enhancing their reproductive output. On the other hand, male size is significantly influenced by sexual selection. During competition for access to females and in the process of fertilization, larger males tend to have a reproductive edge. They may be more successful in out-competing smaller males, either through physical contests or by being more attractive to potential mates [38]. Over the course of evolution, fish have developed a remarkable ability to adapt to their specific habitats and environments. This adaptation is achieved, in part, through the utilization of various sex determination mechanisms and gonadal differentiation patterns. These diverse strategies enable fish to thrive in different ecological niches, ensuring the survival and propagation of their species [39,40]. In some species, the average body size of females is significantly larger than that of males, such as *Salmo gairdneri* [41], *Paralichthys olivaceus* [42] and *Cynoglossus semilaevis* [43], among others. To the contrary, in other species, the average body size of males is greater than that of females, such as *Oreochromis niloticus* [6], *Pelteobagrus fulvidraco* [44], *Ictalurus punctatus* [45] and *Odontobutis obscura* [46]. In this study, we found that sleepy cod belongs to the latter type. At the 12-month-old stage, the growth traits of body weight, body length, body depth and body width for males were 28.27%, 11.56%, 16.54% and 14.35% greater than those for females, respectively. Therefore, all-male sleepy cod breeding is considered valuable, due to the growth potential advantage of males. It is necessary to achieve a high-quality reference transcriptome to rapidly understand the sex determination and gonadal differentiation mechanisms of sleepy cod. Therefore, the high-quality full-length transcriptome of sleepy cod was generated in this study, including 49,113 transcripts with an average length of 3053 bp.

The gonad serves as the primary sex organ. In males, it is composed of the testes while, in females, it is the ovaries. Despite distinct structural differences between genders, the fundamental functions of the gonads remain consistent: they are chiefly responsible for the production of gametes and the secretion of sex hormones. The development and maturation of the gonads involve multiple biological pathways. Previous studies have suggested that sex-biased genes are potentially responsible for phenotypic sexually dimorphic in some aquatic animals [47,48]; however, there have been few genetics-based reports on sex differentiation and gonadal development in sleepy cod. Thus, comparative transcriptomic sequencing was performed in this study in order to reveal some candidate genes. As a result, 18,445 DETs were obtained, including 10,592 ovary-biased transcripts and 8510 testis-biased transcripts. These DETs potentially contribute to gonadal development, gametogenesis, sex determination and differentiation. Well-documented ovary-specific expression genes (*sox19b*, *sox3*, *zar1*, *cpeb1*, *foxl2*, etc.) were identified as ovary-biased genes in this study. Among them, *foxl2* is the first gene that was identified as an ovary-specific expression gene in mammals and plays an vital role in the development and differentiation of the ovaries [49]. Furthermore, testis-specific genes (*dmrt1*, *ar1*, *ar2*, *esr1*, *esr2*, *gsdf*, etc.) have been identified as male-biased genes. The expression of the dmrt1 gene is specifically observed in the testis in mammals, birds and fish, and it is involved in the development and differentiation of the testes [50,51,52,53]. Through protein–protein interaction network analysis involving 20 sex-related genes, a total of 10 proteins were shown to be involved in 30 interactions. The DMRT1 protein formed 7 interaction networks, indicating that dmrt1 genes are a core category affecting sex developmental factors in sleepy cod.

The DETs were assigned with respect to GO categories, and most of the transcripts were involved in DNA helicase activity, ATP binding, 1-SMAD binding, serine-type endopeptidase activity, calcium ion binding and fructose-bisphosphate aldolase activity. These GO terms suggest that the gonadal development of sleepy cod is closely related to sex hormone synthesis and energy metabolism. The KEGG enrichment results showed that oocyte meiosis (ko04114) and arachidonic acid metabolism (ko00590) were the most relevant pathways involved in gonadal differentiation. In this study, more DETs were observed for ovary-biased transcripts (10,592), compared to testis-biased transcripts (8510). This indicates that the metabolism processes in the ovary are complex. In particular, oocyte meiosis is a highly intricate process, which commences with a single round of DNA replication, subsequently succeeded by two rounds of chromosome segregation. Analysis of the oocyte meiosis pathway revealed that the *IGF1R*, *AR*, *SCF*, *Myt1*, *Plkk1*, *SMC1* and SMC3 genes presented testis-bias expression, while *Emil*, *Plk1*, *Emi2*, *Mad1/2*, *PP2A*, *Securin* and *Sgo* genes showed ovary-biased expression. These functional genes may play key roles in gonadal differentiation, and further study is required in this line. Arachidonic acid serves as the precursor for biologically active eicosanoids, including prostaglandins and leukotrienes, which play significant roles in multiple aspects of reproduction [54,55]. In this study, most DETs of the arachidonic acid metabolism pathway were testis-biased, indicating that arachidonic acid may be an important nutrient to ensure the development and maturation of gonads, especially the testis. Studies have shown that, in teleosts such as *Dicentrarchus labrax* [56], *Paralichthys olivaceus* [57], *Hippoglossus hippoglossus* [58] and *Gadus morhua* [59], a diet enriched with higher concentrations of arachidonic acid can significantly enhance fecundity, improve egg quality and increase the viability of larvae. Therefore, the arachidonic acid nutritional requirements should be correspondingly increased in the formulated feed of sleepy cod.

## 5. Conclusions

In summary, we obtained a full-length transcriptome and annotation information as a coding genomic-level reference for sleepy cod. Then, RNA-seq was performed on the testes and ovaries of sleepy cod. A total of 19,102 DETs were identified, of which 8510 transcripts (44.55%) were up-regulated in the ovary and 10,592 transcripts (55.45%) were up-regulated in the testis, respectively. Oocyte meiosis and arachidonic acid metabolism were the most relevant pathways involved in gonadal differentiation. This study yields valuable insights into the genetic mechanisms of sex determination and gonadal differentiation in sleepy cod, providing a potential basis for sex control breeding.

## Figures and Tables

**Table 1:** Primer sequences for qRT-PCR analyses.

Accession ID	Gene	Gene Symbol	Primer Sequence (5′→3′)	Product Length (bp)	Annealing Temperatures (℃)
F01_transcript_17321	SRY-box transcription factor 19b	*sox19b*	F:CCCATATTCACCGGATTCA	118	58
			R:TTCGGATTTCTGCCACTACA		
F01_transcript_19134	SRY-box transcription factor 11b	*sox11b*	F:GCCAAGTCCCTCTAAACCC	141	60
			R:AGTCTGAGGACGGGTCTGTT		
F01_transcript_19661	SRY-box transcription factor 3	*sox3*	F:GTCCTCGGCTCAGACCTAC	120	60
			R:CTTGGTTCACTCTTGCACAC		
F01_transcript_20650	LSM family member 14B	*lsm14b*	F:GGACTGAAGGAAGACTGACTG	121	57
			R:TATTGGGATTGAGGTGGTTC		
F01_transcript_20848	forkhead box H1	*foxh1*	F:TCTGTGGAGGGCAACATAC	124	59
			R:CTGTGATCTGCTGAGTGTCC		
F01_transcript_27462	zygote arrest 1	*zar1*	F:CCTGCACCTGTGAGAAGAA	143	57
			R:TGTCCCATCGCATTATTAGAC		
F01_transcript_32029	cytoplasmic polyadenylation element binding protein 1	*cpeb1*	F:TACTACTGCCGCTCTTGCT	91	58
			R:AATCCCTGTTCTTCTGGTTG		
F01_transcript_39260	forkhead box L2	*foxl2*	F:CCTCACTCTGTCCGGTATCT	147	60
			R:CTGCGGTAGTTTCCCTTCT		
F01_transcript_62040	Gonadotropin releasing hormone receptor 2	*gnrhr2*	F:GGCTTCTACTCTCCGTCCTT	132	60
			R:CATCTCGTGGTCCTCTCTCT		
F01_transcript_69028	zona pellucida glycoprotein 4	*zp4*	F:TTGTTGTTGTCTTGGCTTGT	114	60
			R:GAGAAGTCGTGGTTGAAGGT		
F01_transcript_77894	atrophin-1	*arp*	F:TGACTATGAGAACGGCAGAA	118	57
			R:GTGAAGTGCGTATCCCTGA		
F01_transcript_16742	doublesex- and mab-3-related transcription factor 1	*dmrt1*	F:TATTGTTCTTGGGCTGGTCT	121	60
			R:CCTTTGGATGAGGAGATAGGT		
F01_transcript_31954	doublesex- and mab-3-related transcription factor 3	*dmrt3*	F:TGCTATCGTGGCTCAAAGG	144	60
			R:TCTCCAGACTCTCGTTCGC		
F01_transcript_34248	SRY-box transcription factor 9	*sox9*	F:AAGCTGGGTTTAGAGGGTTT	134	59
			R:TCTTGTTCGTCCGTCATCT		
F01_transcript_39344	androgen receptor-1	*ar1*	F:GATACCAACCAACGAGCAA	85	59
			R:AGCAAGCGTAGTGTCCAAA		
F01_transcript_40103	androgen receptor-2	*ar2*	F:GATACCAACCAACGAGCAA	85	57
			R:AGCAAGCGTAGTGTCCAAA		
F01_transcript_4821	estrogen receptor beta	*esr2*	F:AAGAACGCAGAAAGTGAACC	199	58
			R:TGGAGCAAACACAGAGAGAA		
F01_transcript_60594	estrogen receptor	*esr1*	F:AATGTCCTCTCCAAGTGTCC	143	59
			R:ATGATGGCTGTCTCCCTTT		
F01_transcript_72802	anti-muellerian hormone receptor type-2	*amhr2*	F:TGACTTTGTGCCAGTCTTTATC	94	59
			R:GGTTGTCTACATTGGATGCTATT		
F01_transcript_73590	gonadal soma derived factor	*gsdf*	F:CTCTGTTCCTGATGGTGGA	123	59
			R:GTAATAGTGAGGCTGTTGGGA		
F01_transcript_17112	actin beta	*β-actin*	F:ATCTGGCATCACACCTTCTAC	103	59
			R:TCTTCTCCCTGTTGGCTTT		

Note: F, Forward primer; R, Reverse primer.

**Table 2:** Statistics of growth data of 12-month-old female and male sleepy cod.

Sex	Trait Parameter	Body Weight/g	Body Length/cm	Body Height/cm	Body Width/cm
Female	Mean ± SD	144.85 ± 33.10	18.76 ± 1.72	4.80 ± 0.50	4.67 ± 0.46
	Minmum value	131.30	18.22	4.60	4.50
	Maximum value	158.40	19.30	5.00	4.84
Male	Mean ± SD	185.80 ± 57.29	20.93 ± 1.96	5.60 ± 0.85	5.34 ± 0.71
	Minmum value	171.62	20.37	5.39	5.16
	Maximum value	199.98	21.49	5.81	5.52

Note: SD, Standard deviation.

**Table 3:** The statistics of full-length transcript annotation performed with eight different databases.

Database	Transcripts Annotated Number	Annotated Rate (%)
Transcript number	49,113	
NR	45,361	92.36
eggNOG	44,443	90.49
GO	31,310	63.75
KEGG	30,523	62.15
Pfam	39,448	80.32
Swiss-Prot	31,149	63.42
KOG	33,141	67.48
COG	14,190	28.89
Total	45,505	92.65

**Table 4:** Evaluation statistics of sequencing data.

Sample	Read Number	Base Number	GC Content(%)	≥Q30 (%)
F1	32,572,508	9,744,243,016	49.71	94.94
F2	33,770,463	10,099,245,068	49.95	95.20
F3	31,440,309	9,401,798,866	49.35	95.55
M1	26,243,758	7,852,416,722	48.44	93.14
M2	29,710,081	8,885,414,560	48.75	93.23
M3	30,980,070	9,250,954,850	48.62	93.73

**Table 5:** Annotated statistics of differentially expressed transcripts (DETs).

Annotated	COG	GO	KEGG	KOG	Pfam	Swissprot	eggNOG	NR
18,445	5890	12,575	12,099	13,402	16,570	12,804	18,074	18,401

**Table 6:** Top 20 enriched KEGG pathways in the gonad DETs of sleepy cod.

Pathway	Ko Id	Rich Factor	*q*-Value	Gene Number
Oocyte meiosis	ko04114	1.23	0.0005	156
Arachidonic acid metabolism	ko00590	1.54	0.0007	37
Fatty acid metabolism	ko01212	1.30	0.0023	78
Hedgehog signaling pathway	ko04340	1.34	0.0028	61
Cell adhesion molecules (CAMs)	ko04514	1.17	0.0029	194
Purine metabolism	ko00230	1.16	0.0036	201
ECM-receptor interaction	ko04512	1.27	0.0042	78
Fatty acid elongation	ko00062	1.40	0.0049	40
Spliceosome	ko03040	1.15	0.0049	204
Inositol phosphate metabolism	ko00562	1.21	0.0060	114
Phosphatidylinositol signaling system	ko04070	1.16	0.0113	148
Glycosphingolipid biosynthesis—lacto and neolacto series	ko00601	1.41	0.0119	31
Lysine degradation	ko00310	1.22	0.0132	83
MAPK signaling pathway	ko04010	1.11	0.0136	280
p53 signaling pathway	ko04115	1.18	0.0155	110
RNA polymerase	ko03020	1.36	0.0214	31
Cytokine-cytokine receptor interaction	ko04060	1.15	0.0215	138
Cytosolic DNA-sensing pathway	ko04623	1.30	0.0257	39
Glycerophospholipid metabolism	ko00564	1.14	0.0404	117
Fatty acid biosynthesis	ko00061	1.29	0.0456	31

## Data Availability

The datasets generated and analyzed during this study have been deposited in the Short Read Archive (SRA, http://www.ncbi.nlm.nih.gov/Traces/sra, accessed on 20 February 2023) of the National Center for Biotechnology Information (NCBI) with accession number PRJNA936645.

## Supplementary Materials

The following supporting information can be downloaded at: https://www.mdpi.com/article/10.3390/biology14030232/s1, Table S1: KEGG pathways enriched in the DETs between ovary and testis; Figure S1: The top 20 molecular function categories of GO terms enrichment analysis for the ovary-biased transcripts (A) and the testis-biased transcripts (B), respectively; Figure S2: The top 20 KEGG pathways enriched in the higher-expression genes in the ovary-biased transcripts (A) and the testis-biased transcripts (B), respectively; Figure S3: qRT-PCR analysis of *sox19b* (A), *sox11b* (B), *sox3* (C), *lsm14b* (D), *foxh1* (E), *zar1* (F), *cpeb1* (G), *foxl2* (H), *gnrhr2* (I), *zp4* (J), *arp* (K), *dmrt1* (L), *dmrt3* (M), *sox9* (N), *ar1* (O), *ar2* (P), *esr1* (Q), *esr2* (R), *amhr2* (S), and *gsdf* (T) expression levels in 8 tissues of sleepy cod (Female: *n* = 3; Male: *n* = 3).
