# Supplementary material for: Full-Length Transcriptome Sequencing and Comparative Transcriptomics Reveal the Molecular Mechanisms Underlying Gonadal Development in Sleepy Cod (Oxyeleotris lineolata)
Source: Biology (Basel). 2025 Feb 25;14(3):232. doi: 10.3390/biology14030232 (PMC11940265; doi:10.3390/biology14030232)
Supplement: Supplementary file 1 [file biology-14-00232-s001.zip › biology-3468242-supplementary.pdf]

Supplementary material

Table S1. KEGG pathways enriched in the DETs between ovary and testis.

Figure S1. The top 20 molecular function categories in GO term enrichment analysis for the ovary- (A) and testis-biased transcripts (B).

Figure S2. The top 20 KEGG pathways enriched in the high-expression genes in the ovary- (A) and testis-biased transcripts (B).

Figure S3. qRT-PCR analysis of *sox19b* (A), *sox11b* (B), *sox3* (C), *lsm14b* (D), *foxh1* (E), *zar1* (F), *cpeb1* (G), *foxl2* (H), *gnrhr2* (I), *zp4* (J), *arp* (K), *dmrt1* (L), *dmrt3* (M), *sox9* (N), *ar1* (O), *ar2* (P), *esr1* (Q), *esr2* (R), *amhr2* (S), and *gsdf* (T) expression levels in 8 tissues of sleepy cod (Female: n = 3; Male: n=3).

Table S1. KEGG pathways enriched in the DETs between ovary and testis.

Number	KEGG_pathway	ko_id	*P*-value	All genes number	DETs number
1	Oocyte meiosis	ko04114	0.0005	333	156
2	Arachidonic acid metabolism	ko00590	0.0007	63	37
3	Fatty acid metabolism	ko01212	0.0023	158	78
4	Hedgehog signaling pathway	ko04340	0.0028	120	61
5	Cell adhesion molecules (CAMs)	ko04514	0.0029	436	194
6	Purine metabolism	ko00230	0.0036	455	201
7	ECM-receptor interaction	ko04512	0.0042	161	78
8	Fatty acid elongation	ko00062	0.0049	75	40
9	Spliceosome	ko03040	0.0049	465	204
10	Inositol phosphate metabolism	ko00562	0.0060	248	114
11	Phosphatidylinositol signaling system	ko04070	0.0113	335	148
12	Glycosphingolipid biosynthesis - lacto and neolacto series	ko00601	0.0119	58	31
13	Lysine degradation	ko00310	0.0132	179	83
14	MAPK signaling pathway	ko04010	0.0136	664	280
15	p53 signaling pathway	ko04115	0.0155	245	110
16	RNA polymerase	ko03020	0.0214	60	31
17	Cytokine-cytokine receptor interaction	ko04060	0.0215	316	138
18	Cytosolic DNA-sensing pathway	ko04623	0.0257	79	39
19	Glycerophospholipid metabolism	ko00564	0.0404	270	117
20	Fatty acid biosynthesis	ko00061	0.0456	63	31
21	Linoleic acid metabolism	ko00591	0.0482	27	15
22	Glutathione metabolism	ko00480	0.0489	101	47
23	Biosynthesis of unsaturated fatty acids	ko01040	0.0490	61	30
24	Dorso-ventral axis formation	ko04320	0.0511	87	41
25	Aminoacyl-tRNA biosynthesis	ko00970	0.0588	131	59
26	Metabolism of xenobiotics by cytochrome P450	ko00980	0.0599	76	36
27	N-Glycan biosynthesis	ko00510	0.0601	136	61
28	Pantothenate and CoA biosynthesis	ko00770	0.0625	19	11
29	Fatty acid degradation	ko00071	0.0673	149	66
30	Vascular smooth muscle contraction	ko04270	0.0709	313	132
31	alpha-Linolenic acid metabolism	ko00592	0.0887	38	19
32	Ether lipid metabolism	ko00565	0.0945	110	49
33	AGE-RAGE signaling pathway in diabetic complications	ko04933	0.1045	358	148
34	Ubiquitin mediated proteolysis	ko04120	0.1102	417	171
35	Glycosaminoglycan biosynthesis - chondroitin sulfate / dermatan sulfate	ko00532	0.1129	32	16
36	beta-Alanine metabolism	ko00410	0.1144	70	32
37	Pyrimidine metabolism	ko00240	0.1151	281	117
38	Other glycan degradation	ko00511	0.1273	49	23
39	Phototransduction	ko04744	0.1411	19	10
40	D-Arginine and D-ornithine metabolism	ko00472	0.1445	2	2
41	TGF-beta signaling pathway	ko04350	0.1484	216	90
42	Cell cycle	ko04110	0.1505	455	184
43	Lysine biosynthesis	ko00300	0.1571	4	3
44	Sulfur metabolism	ko00920	0.1585	24	12
45	VEGF signaling pathway	ko04370	0.1629	207	86
46	Sphingolipid metabolism	ko00600	0.1779	125	53
47	Cardiac muscle contraction	ko04260	0.1879	191	79
48	Mismatch repair	ko03430	0.1924	66	29
49	Glycosaminoglycan biosynthesis - heparan sulfate / heparin	ko00534	0.2010	54	24
50	SNARE interactions in vesicular transport	ko04130	0.2084	74	32
51	Taurine and hypotaurine metabolism	ko00430	0.2086	18	9
52	Progesterone-mediated oocyte maturation	ko04914	0.2150	310	125
53	ABC transporters	ko02010	0.2227	82	35
54	Propanoate metabolism	ko00640	0.2228	87	37
55	Adrenergic signaling in cardiomyocytes	ko04261	0.2229	439	175
56	GnRH signaling pathway	ko04912	0.2435	271	109
57	Glycosaminoglycan degradation	ko00531	0.2492	48	21
58	Steroid hormone biosynthesis	ko00140	0.2685	51	22
59	Valine, leucine and isoleucine biosynthesis	ko00290	0.2837	5	3

60	Glycosphingolipid biosynthesis - ganglio series	ko00604	0.2872	39	17
61	RNA degradation	ko03018	0.3084	275	109
62	Glycosaminoglycan biosynthesis - keratan sulfate	ko00533	0.3099	37	16
63	Rheumatoid arthritis	ko05323	0.3236	3	2
64	African trypanosomiasis	ko05143	0.3236	3	2
65	Antigen processing and presentation	ko04612	0.3236	3	2
66	Glycosphingolipid biosynthesis - globo series	ko00603	0.3359	25	11
67	Gap junction	ko04540	0.3481	241	95
68	Ras signaling pathway	ko04014	0.3676	13	6
69	Prion diseases	ko05020	0.3801	1	1
70	Basal transcription factors	ko03022	0.3945	98	39
71	RIG-I-like receptor signaling pathway	ko04622	0.4038	145	57
72	T cell receptor signaling pathway	ko04660	0.4146	6	3
73	Natural killer cell mediated cytotoxicity	ko04650	0.4146	6	3
74	Sulfur relay system	ko04122	0.4406	19	8
75	Drug metabolism - other enzymes	ko00983	0.4445	97	38
76	Aldosterone synthesis and secretion	ko04925	0.4523	14	6
77	Terpenoid backbone biosynthesis	ko00900	0.4653	48	19
78	Notch signaling pathway	ko04330	0.4721	166	64
79	Other types of O-glycan biosynthesis	ko00514	0.4762	98	38
80	Valine, leucine and isoleucine degradation	ko00280	0.4777	140	54
81	Huntington's disease	ko05016	0.4902	4	2
82	Fc epsilon RI signaling pathway	ko04664	0.4902	4	2
83	Glycerolipid metabolism	ko00561	0.5041	191	73
84	Alzheimer's disease	ko05010	0.5047	12	5
85	ErbB signaling pathway	ko04012	0.5078	307	117
86	Retinol metabolism	ko00830	0.5203	73	28
87	One carbon pool by folate	ko00670	0.5230	44	17
88	Mucin type O-Glycan biosynthesis	ko00512	0.5309	68	26
89	cGMP-PKG signaling pathway	ko04022	0.5340	15	6
90	Glioma	ko05214	0.5362	7	3
91	Insulin secretion	ko04911	0.5362	7	3
92	Chagas disease (American trypanosomiasis)	ko05142	0.5668	10	4
93	Pertussis	ko05133	0.5668	10	4
94	PPAR signaling pathway	ko03320	0.5712	247	93
95	FoxO signaling pathway	ko04068	0.5771	512	193
96	Protein export	ko03060	0.5818	56	21
97	Neurotrophin signaling pathway	ko04722	0.5904	13	5
98	Estrogen signaling pathway	ko04915	0.5904	13	5
99	Long-term potentiation	ko04720	0.5904	13	5
100	Inflammatory mediator regulation of TRP channels	ko04750	0.5904	13	5
101	Amino sugar and nucleotide sugar metabolism	ko00520	0.5904	139	52
102	Salivary secretion	ko04970	0.6099	16	6
103	Chronic myeloid leukemia	ko05220	0.6158	2	1
104	Staphylococcus aureus infection	ko05150	0.6158	2	1
105	Complement and coagulation cascades	ko04610	0.6158	2	1
106	Longevity regulating pathway - worm	ko04212	0.6279	5	2
107	Fc gamma R-mediated phagocytosis	ko04666	0.6279	5	2
108	Endocrine and other factor-regulated calcium reabsorption	ko04961	0.6279	5	2
109	Osteoclast differentiation	ko04380	0.6279	5	2
110	Pancreatic cancer	ko05212	0.6279	5	2
111	Folate biosynthesis	ko00790	0.6412	33	12
112	Olfactory transduction	ko04740	0.6418	8	3
113	Retrograde endocannabinoid signaling	ko04723	0.6418	8	3
114	Cholinergic synapse	ko04725	0.6418	8	3
115	Ribosome biogenesis in eukaryotes	ko03008	0.6485	219	81
116	Galactose metabolism	ko00052	0.6490	101	37
117	Renin secretion	ko04924	0.6671	14	5
118	Wnt signaling pathway	ko04310	0.6681	439	163
119	Endocytosis	ko04144	0.6755	1056	395
120	Calcium signaling pathway	ko04020	0.6817	472	175
121	Butanoate metabolism	ko00650	0.7127	54	19
122	Primary bile acid biosynthesis	ko00120	0.7164	29	10
123	Systemic lupus erythematosus	ko05322	0.7291	9	3
124	Sphingolipid signaling pathway	ko04071	0.7301	12	4
125	Selenocompound metabolism	ko00450	0.7321	49	17

126	Toll-like receptor signaling pathway	ko04620	0.7329	229	83
127	Glucagon signaling pathway	ko04922	0.7337	15	5
128	Circadian entrainment	ko04713	0.7337	15	5
129	Gastric acid secretion	ko04971	0.7337	15	5
130	Amoebiasis	ko05146	0.7346	6	2
131	Thyroid hormone synthesis	ko04918	0.7346	6	2
132	Riboflavin metabolism	ko00740	0.7346	6	2
133	Glycosylphosphatidylinositol(GPI)-anchor biosynthesis	ko00563	0.7397	38	13
134	Leishmaniasis	ko05140	0.7619	3	1
135	Choline metabolism in cancer	ko05231	0.7619	3	1
136	Fat digestion and absorption	ko04975	0.7619	3	1
137	Fructose and mannose metabolism	ko00051	0.7630	152	54
138	Steroid biosynthesis	ko00100	0.7662	33	11
139	Arginine and proline metabolism	ko00330	0.7731	158	56
140	Base excision repair	ko03410	0.7846	87	30
141	Nucleotide excision repair	ko03420	0.7863	112	39
142	Alcoholism	ko05034	0.7899	16	5
143	Pathways in cancer	ko05200	0.7900	19	6
144	Drug metabolism - cytochrome P450	ko00982	0.8076	74	25
145	mRNA surveillance pathway	ko03015	0.8092	283	101
146	Serotonergic synapse	ko04726	0.8140	7	2
147	Transcriptional misregulation in cancer	ko05202	0.8140	7	2
148	Intestinal immune network for IgA production	ko04672	0.8162	94	32
149	RNA transport	ko03013	0.8219	525	190
150	Phenylalanine, tyrosine and tryptophan biosynthesis	ko00400	0.8321	26	8
151	Apoptosis	ko04210	0.8325	483	174
152	Vitamin B6 metabolism	ko00750	0.8364	17	5
153	D-Glutamine and D-glutamate metabolism	ko00471	0.8364	17	5
154	Dopaminergic synapse	ko04728	0.8364	17	5
155	Tuberculosis	ko05152	0.8422	14	4
156	Ascorbate and aldarate metabolism	ko00053	0.8523	56	18
157	Non-alcoholic fatty liver disease (NAFLD)	ko04932	0.8524	4	1
158	Renal cell carcinoma	ko05211	0.8524	4	1
159	B cell receptor signaling pathway	ko04662	0.8524	4	1
160	Lipoic acid metabolism	ko00785	0.8524	4	1
161	Measles	ko05162	0.8524	4	1
162	Thyroid cancer	ko05216	0.8524	4	1
163	Axon guidance	ko04360	0.8525	11	3
164	Amphetamine addiction	ko05031	0.8525	11	3
165	Regulation of autophagy	ko04140	0.8548	59	19
166	Ribosome	ko03010	0.8588	238	83
167	Adipocytokine signaling pathway	ko04920	0.8623	285	100
168	AMPK signaling pathway	ko04152	0.8714	8	2
169	Long-term depression	ko04730	0.8714	8	2
170	cAMP signaling pathway	ko04024	0.8741	18	5
171	NOD-like receptor signaling pathway	ko04621	0.8808	140	47
172	Pancreatic secretion	ko04972	0.8816	15	4
173	Cysteine and methionine metabolism	ko00270	0.8858	174	59
174	Peroxisome	ko04146	0.8866	249	86
175	Tryptophan metabolism	ko00380	0.8903	104	34
176	Biotin metabolism	ko00780	0.8933	12	3
177	Pentose phosphate pathway	ko00030	0.8944	110	36
178	Focal adhesion	ko04510	0.8949	711	255
179	Glycine, serine and threonine metabolism	ko00260	0.9003	119	39
180	Proteasome	ko03050	0.9034	105	34
181	Colorectal cancer	ko05210	0.9085	5	1
182	Vasopressin-regulated water reabsorption	ko04962	0.9085	5	1
183	Non-homologous end-joining	ko03450	0.9141	29	8
184	Nicotinate and nicotinamide metabolism	ko00760	0.9179	112	36
185	Pyruvate metabolism	ko00620	0.9222	141	46
186	Pentose and glucuronate interconversions	ko00040	0.9266	78	24
187	Starch and sucrose metabolism	ko00500	0.9335	128	41
188	Viral carcinogenesis	ko05203	0.9355	17	4
189	Tyrosine metabolism	ko00350	0.9365	73	22
190	Citrate cycle (TCA cycle)	ko00020	0.9397	123	39
191	Melanogenesis	ko04916	0.9404	301	102

192	Lysosome	ko04142	0.9407	445	154
193	Oxidative phosphorylation	ko00190	0.9410	276	93
194	HTLV-I infection	ko05166	0.9433	6	1
195	2-Oxocarboxylic acid metabolism	ko01210	0.9549	72	21
196	Histidine metabolism	ko00340	0.9593	48	13
197	Glutamatergic synapse	ko04724	0.9599	11	2
198	Ubiquinone and other terpenoid-quinone biosynthesis	ko00130	0.9599	22	5
199	Insulin signaling pathway	ko04910	0.9630	490	168
200	Regulation of actin cytoskeleton	ko04810	0.9640	716	250
201	Caffeine metabolism	ko00232	0.9649	7	1
202	Rap1 signaling pathway	ko04015	0.9685	33	8
203	mTOR signaling pathway	ko04150	0.9731	483	164
204	Thiamine metabolism	ko00730	0.9732	12	2
205	HIF-1 signaling pathway	ko04066	0.9732	12	2
206	Chemokine signaling pathway	ko04062	0.9732	12	2
207	Viral myocarditis	ko05416	0.9735	16	3
208	Homologous recombination	ko03440	0.9789	73	20
209	Carbon metabolism	ko01200	0.9805	445	149
210	Biosynthesis of amino acids	ko01230	0.9811	347	114
211	Oxytocin signaling pathway	ko04921	0.9812	28	6
212	Butirosin and neomycin biosynthesis	ko00524	0.9827	21	4
213	Leukocyte transendothelial migration	ko04670	0.9827	21	4
214	Synthesis and degradation of ketone bodies	ko00072	0.9827	21	4
215	DNA replication	ko03030	0.9860	136	40
216	Phospholipase D signaling pathway	ko04072	0.9865	9	1
217	Herpes simplex infection	ko05168	0.9867	805	277
218	Platelet activation	ko04611	0.9878	22	4
219	Neuroactive ligand-receptor interaction	ko04080	0.9879	340	110
220	Porphyrin and chlorophyll metabolism	ko00860	0.9906	105	29
221	Phenylalanine metabolism	ko00360	0.9906	51	12
222	Nitrogen metabolism	ko00910	0.9909	61	15
223	Arginine biosynthesis	ko00220	0.9916	124	35
224	Legionellosis	ko05134	0.9916	10	1
225	Fanconi anemia pathway	ko03460	0.9952	124	34
226	Bacterial invasion of epithelial cells	ko05100	0.9967	17	2
227	Alanine, aspartate and glutamate metabolism	ko00250	0.9973	161	45
228	Proteoglycans in cancer	ko05205	0.9980	27	4
229	Arrhythmogenic right ventricular cardiomyopathy (ARVC)	ko05412	0.9980	13	1
230	Adherens junction	ko04520	0.9983	515	165
231	Protein processing in endoplasmic reticulum	ko04141	0.9987	532	170
232	Thyroid hormone signaling pathway	ko04919	0.9988	14	1
233	Glyoxylate and dicarboxylate metabolism	ko00630	0.9989	122	31
234	Hypertrophic cardiomyopathy (HCM)	ko05410	0.9991	20	2
235	Dilated cardiomyopathy	ko05414	0.9996	22	2
236	Glycolysis / Gluconeogenesis	ko00010	0.9997	286	82
237	Influenza A	ko05164	0.9998	28	3
238	Salmonella infection	ko05132	0.9999	274	75
239	Hippo signaling pathway	ko04390	1.0000	21	1
240	Phagosome	ko04145	1.0000	723	221
241	Tight junction	ko04530	1.0000	639	185

A

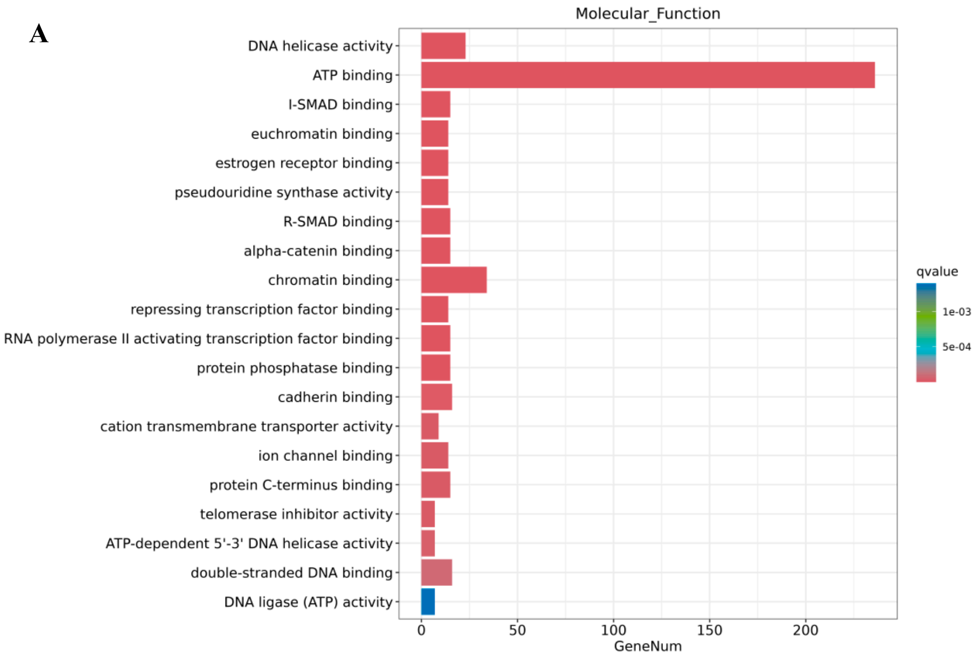

B

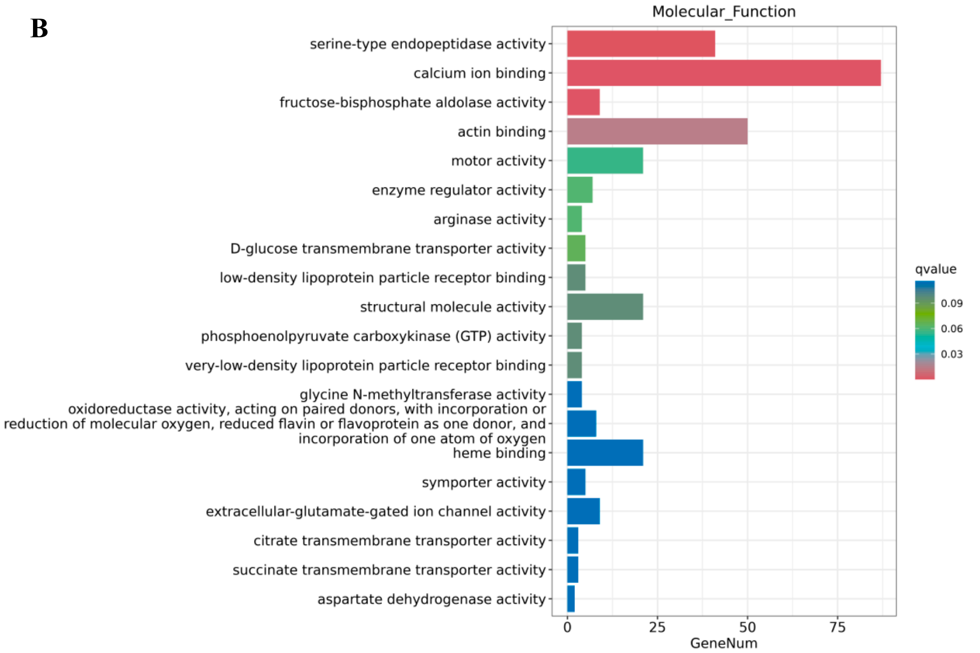

Figure S1. The top 20 molecular function categories in GO term enrichment analysis for the ovary- (A) and testis-biased transcripts (B).

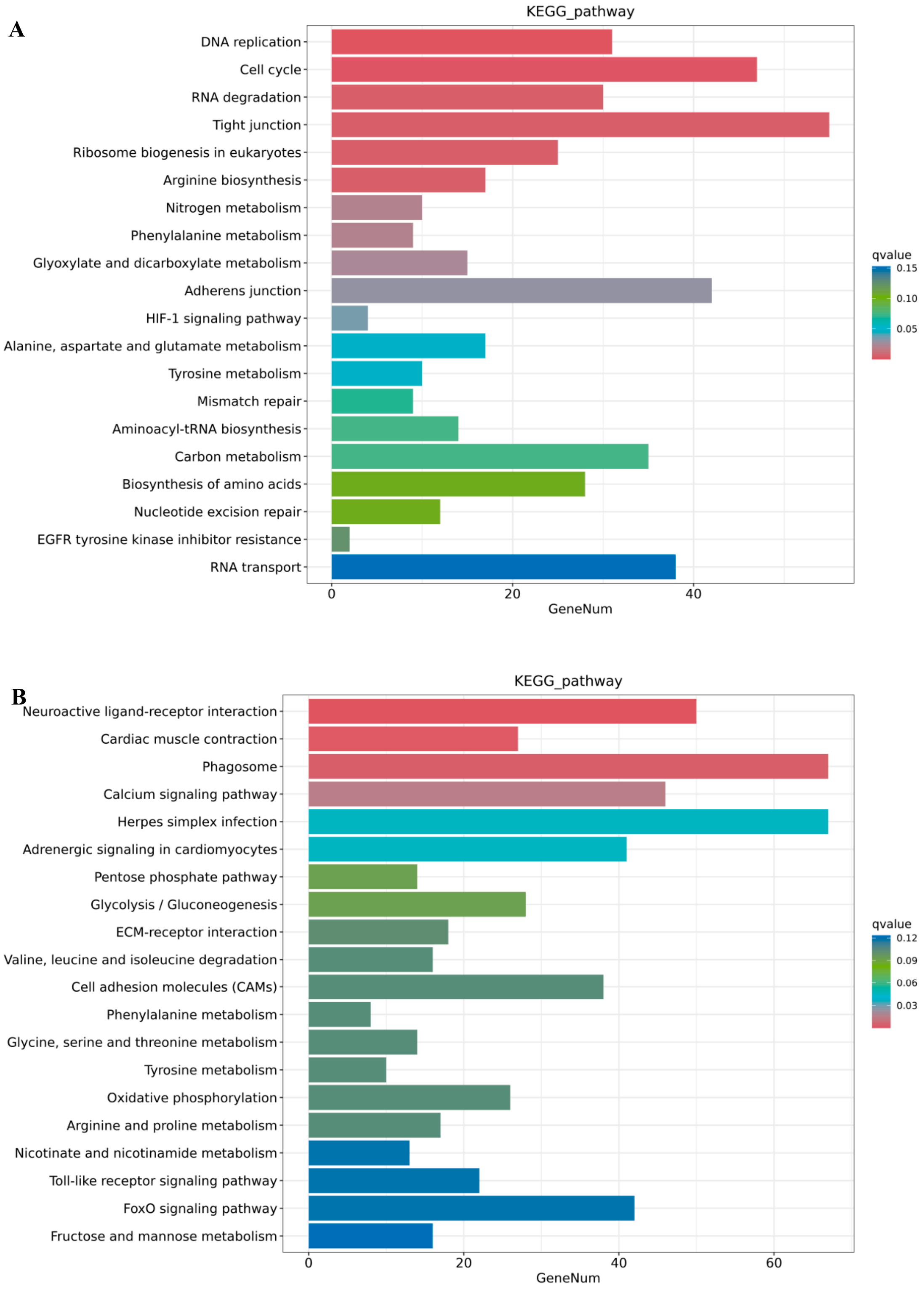

Figure S2. The top 20 KEGG pathways enriched in the high-expression genes in the ovary- (A) and testis-biased transcripts (B).

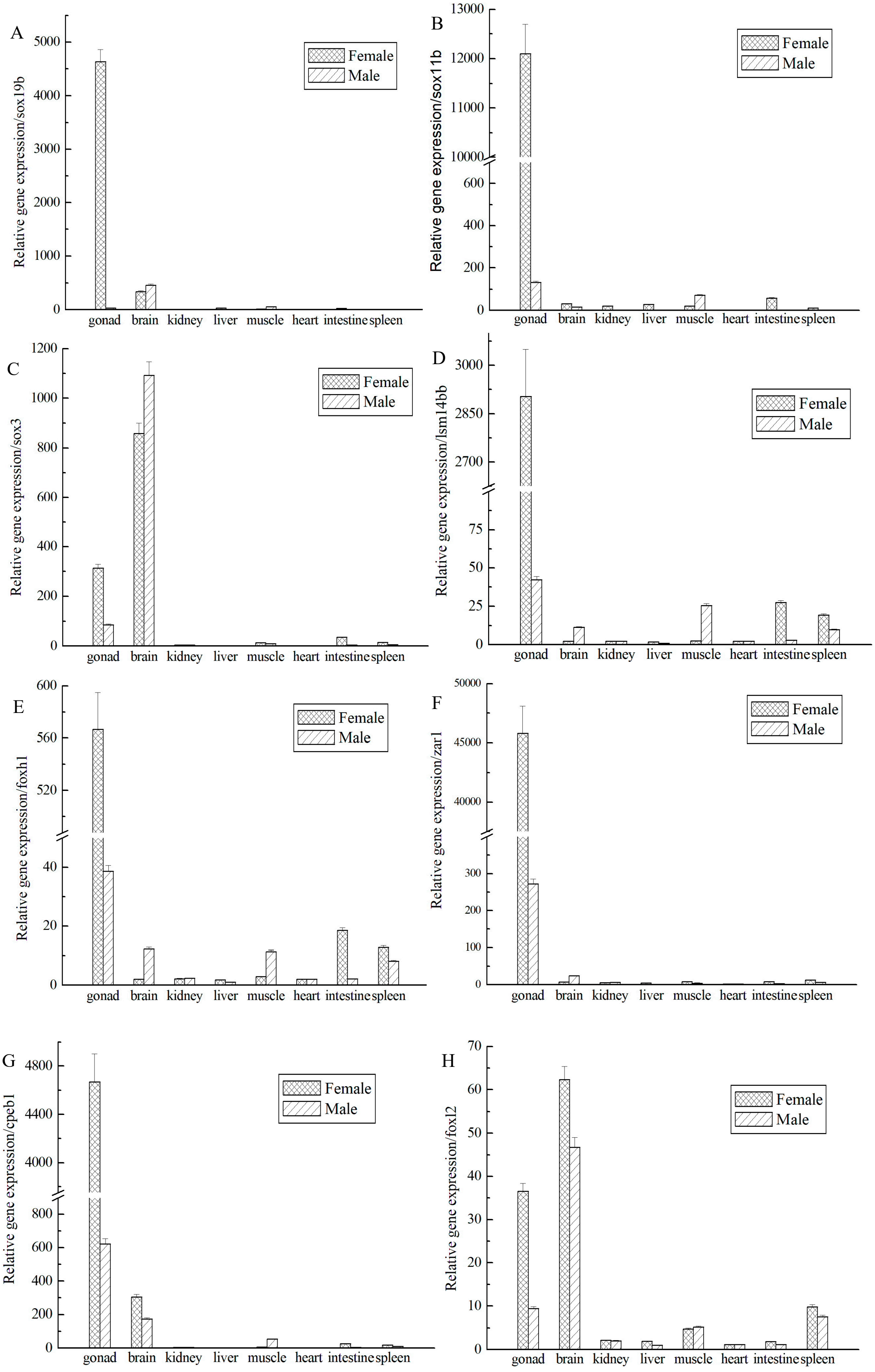
A
Relative gene expression/sox19b
0
1000
2000
3000
4000
5000
Female
Male
gonad brain kidney liver muscle heart intestine spleen
B
Relative gene expression/sox11b
0
200
400
600
10000
11000
12000
13000
Female
Male
gonad brain kidney liver muscle heart intestine spleen
C
Relative gene expression/sox3
0
200
400
600
800
1000
1200
Female
Male
gonad brain kidney liver muscle heart intestine spleen
D
Relative gene expression/lsm14bb
0
25
50
75
2700
2850
3000
Female
Male
gonad brain kidney liver muscle heart intestine spleen
E
Relative gene expression/foxh1
0
20
40
520
560
600
Female
Male
gonad brain kidney liver muscle heart intestine spleen
F
Relative gene expression/zar1
0
100
200
300
40000
45000
50000
Female
Male
gonad brain kidney liver muscle heart intestine spleen
G
Relative gene expression/cpeb1
0
200
400
600
800
4000
4400
4800
Female
Male
gonad brain kidney liver muscle heart intestine spleen
H
Relative gene expression/foxl2
0
10
20
30
40
50
60
70
Female
Male
gonad brain kidney liver muscle heart intestine spleen

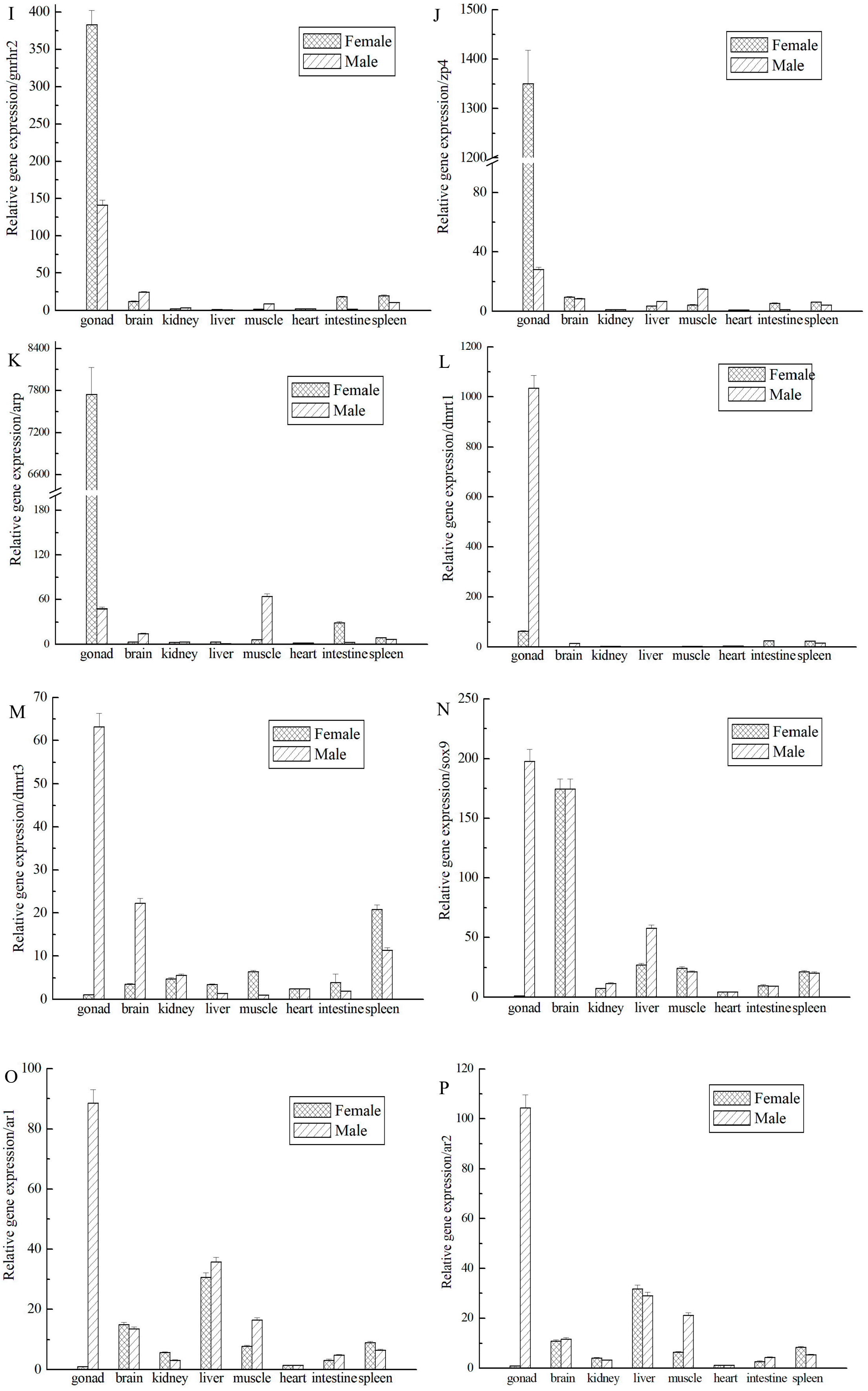
I
Relative gene expression/gnrhr2
0
50
100
150
200
250
300
350
400
Female
Male
gonad brain kidney liver muscle heart intestine spleen
J
Relative gene expression/zp4
0
40
80
1200
1300
1400
1500
Female
Male
gonad brain kidney liver muscle heart intestine spleen
K
Relative gene expression/arp
0
60
120
180
6600
7200
7800
8400
Female
Male
gonad brain kidney liver muscle heart intestine spleen
L
Relative gene expression/dmrt1
0
200
400
600
800
1000
1200
Female
Male
gonad brain kidney liver muscle heart intestine spleen
M
Relative gene expression/dmrt3
0
10
20
30
40
50
60
70
Female
Male
gonad brain kidney liver muscle heart intestine spleen
N
Relative gene expression/sox9
0
50
100
150
200
250
Female
Male
gonad brain kidney liver muscle heart intestine spleen
O
Relative gene expression/ar1
0
20
40
60
80
100
Femal
Male
gonad brain kidney liver muscle heart intestine spleen
P
Relative gene expression/ar2
0
20
40
60
80
100
120
Female
Male
gonad brain kidney liver muscle heart intestine spleen

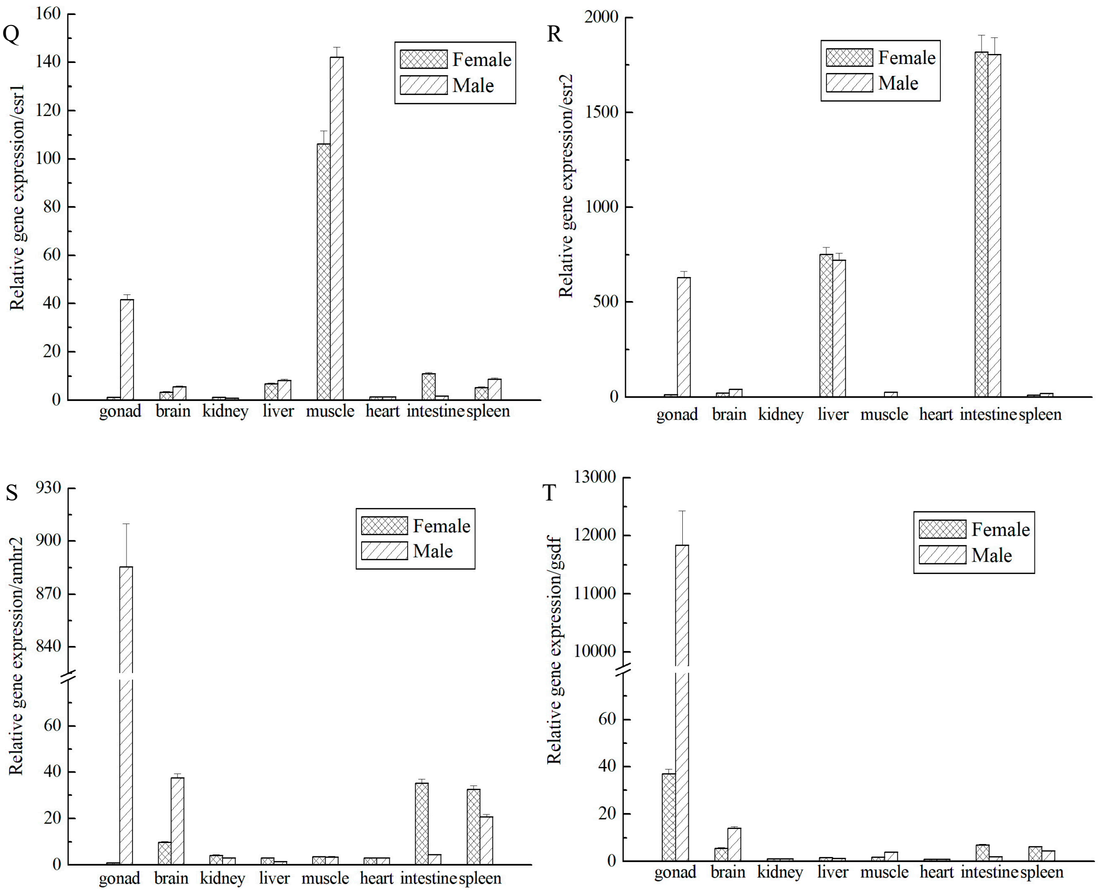

Figure S3. qRT-PCR analysis of *sox19b* (A), *sox11b* (B), *sox3* (C), *lsm14b* (D), *foxh1* (E), *zar1* (F), *cpeb1* (G), *foxl2* (H), *gnrhr2* (I), *zp4* (J), *arp* (K), *dmrt1* (L), *dmrt3* (M), *sox9* (N), *ar1* (O), *ar2* (P), *esr1* (Q), *esr2* (R), *amhr2* (S), and *gsdf* (T) expression levels in 8 tissues of sleepy cod (Female: n = 3; Male: n=3).
